# Investigation of Fats, Oils, and Grease Co-digestion With Food Waste in Anaerobic Membrane Bioreactors and the Associated Microbial Community Using MinION Sequencing

**DOI:** 10.3389/fbioe.2021.613626

**Published:** 2021-04-12

**Authors:** Syeed Md Iskander, Yamrot M. Amha, Phillip Wang, Qin Dong, Juhe Liu, Michael Corbett, Adam L. Smith

**Affiliations:** ^1^Astani Department of Civil and Environmental Engineering, University of Southern California, Los Angeles, CA, United States; ^2^Department of Civil and Environmental Engineering, North Dakota State University, Fargo, ND, United States; ^3^Trussell Technologies, Inc., Pasadena, CA, United States; ^4^Divert, Inc., Concord, MA, United States

**Keywords:** AnMBR, FOG, biogas, MinION sequencing, inhibition

## Abstract

Co-digestion of fats, oils, and grease (FOG) with food waste (FW) can improve the energy recovery in anaerobic membrane bioreactors (AnMBRs). Here, we investigated the effect of co-digestion of FW and FOG in AnMBRs at fat mass loading of 0.5, 0.75, and 1.0 kg m^–3^ day^–1^ with a constant organic loading rate of 5.0 gCOD L^–1^ day^–1^ in both a single-phase (SP) and two-phase (TP) configuration. A separate mono-digestion of FW at an identical organic loading rate was used as the benchmark. During co-digestion, higher daily biogas production, ranging from 4.0 to 12.0%, was observed in the two-phase methane phase (TP-MP) reactor compared to the SP reactor, but the difference was statistically insignificant (*p* > 0.05) due to the high variability in daily biogas production. However, the co-digestion of FW with FOG at 1.0 kg m^–3^ day^–1^ fat loading rate significantly (*p* < 0.05) improved daily biogas production in both the SP (11.0%) and TP (13.0%) reactors compared to the mono-digestion of FW. Microbial community analyses using cDNA-based MinION sequencing of weekly biomass samples from the AnMBRs revealed the prevalence of *Lactobacillus* (92.2–95.7% relative activity) and *Anaerolineaceae* (13.3–57.5% relative activity), which are known as fermenters and fatty acid degraders. Syntrophic fatty acid oxidizers were mostly present in the SP and TP-MP reactors, possibly because of the low pH and short solid retention time (SRT) in the acid phase digesters. A greater abundance of the *mcrA* gene copies (and methanogens) was observed in the SP and MP reactors compared to the acid-phase (AP) reactors. This study demonstrates that FW and FOG can be effectively co-digested in AnMBRs and is expected to inform full-scale decisions on the optimum fat loading rate.

## Introduction

Fats, oils, and grease (FOG) from cooking and food processing industries are generally collected in grease traps and interceptors to prevent damage to sewage collection systems (Alqaralleh et al., 2016; Amha et al., 2017). Management of FOG in anaerobic digesters can reduce environmental impacts by diverting it from landfills (i.e., the conventional method for FOG management), while enabling energy recovery in the form of increased biogas production from the additional substrate (Alqaralleh et al., 2016). Various studies have reported increased energy recovery by co-digesting FOG with other organic waste streams (Long et al., 2012; Amha et al., 2017). Anaerobic membrane bioreactors (AnMBRs), which combine anaerobic digestion and membrane separation to improve effluent quality and increase biogas production, are an attractive biotechnology for organic waste management (Smith et al., 2012; Lin H. J. et al., 2013). Amha et al. (2019) demonstrated that AnMBR management of food waste (FW) provided >95% chemical oxygen demand (COD) removal. Further, AnMBR was reported to produce 0.13–0.18 L CH_4_ per g of COD removed during treatment of food processing wastewater with a net energy benefit of 0.16–1.82 kWh m^–3^ (Galib et al., 2016).

The recent legislative push in the United States (e.g., California, Connecticut, Massachusetts, Rhode Island, and Vermont) and elsewhere to divert organic waste from landfills makes AnMBRs an attractive management strategy for FW and FOG management (Leibrock, 2017). The addition of FOG to AnMBRs can enhance energy recovery by increasing the organic loading rate and potentially compensating for temporal fluctuations in FW characteristics. FOG is more desirable as a substrate for co-digestion because of the higher theoretical biomethane yield (1.0 m^3^ CH_4_ kg^–1^) compared to protein (0.63 m^3^ CH_4_ kg^–1^) and carbohydrate (0.42 m^3^ CH_4_ kg^–1^) (Jeganathan et al., 2006; Xu et al., 2018). A previous study reported an optimum methane production of 800 L (kg VS) ^–1^ during FOG (30% w/w) co-digestion with FW (70% w/w), which was further enhanced by granular activated carbon addition (Chowdhury et al., 2019). We previously reported that the addition of FOG with FW during anaerobic digestion in batch assays at thermophilic conditions led to increased activity of key syntrophic fatty acid-degrading populations that were directly correlated with improved performance (Amha et al., 2017). FW addition increased methane production by 18.4% during anaerobic digestion of primary and waste activated sludge, while FOG addition resulted in an increase of 21.1%. The addition of both FW and FOG resulted in a 26.0% increase in methane production (Amha et al., 2017).

Despite benefits to energy recovery from FOG addition, the addition of FOG can increase the concentration of long-chain fatty acids (LCFAs) that can negatively impact the microbial community by causing damage to cell membranes, reducing nutrient transport, and decreasing cell permeability (Palatsi et al., 2010; Long et al., 2012; Sousa et al., 2013). The accumulation of LCFA on biomass, however, can exert decreased fouling propensity due to increased sludge hydrophobicity, although prior observations on this potential benefit are inconsistent (Ramos et al.,2014a,b; Dereli et al., 2015). LCFA accumulation on sludge can cause flotation and result in poor biomass retention (Alves et al., 2009); however, membrane filtration in AnMBRs minimizes the washout. A full-scale AnMBR operated by Divert Inc. (Compton, CA) operates under a maximum fat loading rate of 0.5 kg m^–3^day^–1^, as their observations beyond this rate have indicated process inhibition and operational concerns (e.g., erratic gas production, fat flotation, and calcium oleate fat sphere formation). Similarly, another study has reported the formation of fat balls at fat loading rates beyond 1.16 kg m^–3^ day^–1^ during lipid-rich corn-to-ethanol thin stillage wastewater treatment (Dereli et al., 2014). Therefore, in this study, we aimed to evaluate FW and FOG co-digestion in AnMBRs in both single-phase (SP) and two-phase (TP) configurations. In the TP configuration, the acid phase and methane phase are separated in two different reactors. In the two-phase acid-phase (TP-AP) reactor, substrate hydrolysis is optimized by maintaining a short hydraulic retention time (HRT), solid retention time (SRT), and low pH (∼3.5). The two-phase methane-phase (TP-MP) reactor is operated at longer HRT, SRT, and neutral pH and is therefore expected to enrich for a more diverse microbial community than the SP. Our previous study on FW management in TP AnMBRs demonstrated a significant increase in methane production compared to SP AnMBRs at ORLs up to 10 gCOD L^–1^d^–1^. The increase, ranging from 15.2 to 20.3%, was a result of increased VFA production (up to three times compared to the influent FW) in the AP of the TP reactor and increased diversity of the microbial community with higher activity of syntrophic fatty acid oxidizers in the TP-MP reactor compared to the SP reactor (Amha et al., 2019). However, the economic feasibility of a TP over a SP AnMBR is yet to be evaluated.

Although there are a few studies that have investigated AnMBRs for high-lipid wastewater (Dereli et al., 2012, 2014, 2015; Jensen et al., 2015), FW and FOG management in AnMBRs has yet to be evaluated. Further, no study to date has characterized the microbial community during high-lipid waste management in AnMBRs. Thus, the objective of this study was to evaluate the co-digestion of FW and FOG in bench-scale SP and TP AnMBRs. We systematically increased FOG addition to investigate the optimum and maximum fat loading rate without negatively affecting system performance and the microbial community. Further, the microbial community response to FOG addition was evaluated using Nanopore MinION sequencing of the 16S rRNA as a metric of microbial activity. The results of this study are expected to advise operation of full-scale AnMBRs during FOG co-digestion or management of other high-lipid waste streams.

## Materials and Methods

### Bench-Scale AnMBR Configurations

Two identical jacketed 7-L reactors (Chemglass, NJ) with 5 L liquid volume were operated in the study (Supplementary Figure 1). Each reactor (SP and TP-MP) contained a submerged flat-sheet ceramic microfiltration membrane with a pore size of 0.1 μm and a total effective membrane area of 0.011 m^2^. Both reactors were mixed continuously at 200 rpm with an impellor located near the bottom of the reactor vessel. The jackets of the reactors were connected to a recirculating water bath (Fisher Scientific, Hampton, NH) to maintain the reactor temperature at 37°C. The temperature in each reactor was monitored via a probe submerged in the mixed liquor. Pressure in the headspace and permeate lines were monitored using pressure transducers (Transducers Direct, Cincinnati, OH). The influent and permeate were pumped using peristaltic pumps to maintain a constant liquid volume in the AnMBRs (New Era, Farmingdale, NY, and Langer, Boonton, NJ). Membrane backwashing was conducted for 3 min during 10-min operational intervals for fouling control. The headspace was connected to a gas mass flow meter (GFM, Aalborg, New York) to measure the biogas flow. To mitigate membrane fouling, a mini diaphragm pump (Parker, North Carolina) recirculated produced biogas at 1 m^3^ m^–2^ h^–1^ through sparging tubes mounted below the membrane housing. All data acquisition and permeate pump controls were conducted with LabVIEW (National Instruments, Austin, TX) data acquisition software that recorded data every minute. The TP-AP reactor was a 1-L glass bottle continuously stirred at 250 rpm in a 37°C water bath. The SRT and HRT of the rector were maintained at 3 days. The details on the AnMBR configuration can be found in literature (Amha et al., 2019).

### Inoculation and Operational Parameters

Both the SP and TP AnMBRs were inoculated with mixed liquor from lab-scale SP and TP mesophilic (37°C) AnMBRs treating FW. For the TP configuration, both the TP-AP and TP-MP were inoculated with mixed liquor from TP-AP and TP-MP reactors, respectively. The SP inocula had 43.0 g L^–1^ total solids (TS) and 35.0 g L^–1^ total volatile solids (TVS), the TP-AP inocula had 37.0 g L^–1^ TS and 31.0 g L^–1^ TVS, and the TP-MP had 46.0 g L^–1^ TS and 36.6 g L^–1^ TVS. FW was sieved using a 1-mm aluminum mesh to remove large particulates that would otherwise clog reactor tubing. The sieved feed FW had an average chemical oxygen demand (COD) of 123 g L^–1^, TS of 65.8 g L^–1^, TVS of 60.6 g L^–1^, pH of 3.50, and NH_4_-N of 1.92 g L^–1^ and was collected from Divert, Inc. (Compton, CA, United States). To prevent biodegradation, the feed was kept at 4°C during operation of the reactors. The FOG had a COD of 391 g L^–1^, pH of 4.26, TS of 534 g L^–1^, and TVS of 528 g L^–1^ and was collected from Baker Commodities (Vernon, CA), which collects and manages FOG retrieved from restaurants. Fats were analyzed using the acid hydrolysis method that targets all fatty acids, triglycerides, esters, long-chain alcohols, hydrocarbons, and other glycol esters and sterols (Ziels et al., 2015; Ngeh-Ngwainbi et al., 2020). First, 10 mL of 4.0 N hydrochloric acid and 2 mL of 95% (v/v) ethanol were added to 10 g FOG sample and incubated at 70°C for 40 min. Next, crude fats were extracted using diethyl and petroleum ether solvents. The solvent addition was repeated in four cycles with 40 mL diethyl and 40 mL petroleum ether in each cycle to ensure complete extraction of fats. The dry weight (60°C for 24 h) of FOG samples was measured before and after fat extraction to quantify the fat weight percent. The operational parameters of the AnMBR are summarized in Table 1.

**TABLE 1:** Operating conditions of the SP and TP-MP AnMBRs.

Operating conditions	Value	Unit
OLR	5 ± 0.03	g COD L^–1^ day^–1^
Temperature	37 ± 0.1	°C
HRT	26–42	Day
SRT	100 ± 0.04	Day
Membrane area	0.011	m^2^
Permeate flux	0.5–0.8	L m^–2^ h^–1^
Reactor volume	5.2	L
FW COD	123 ± 10.5	g L^–1^
Biogas sparging	1	m^3^ m^–2^ h^–1^
Backwash	3 min per 10 min	
		

During 0.5–0.75 kg m^–3^ day^–1^ fat loading rates, the SP and TP-AP reactors were operated with a mixed FW and FOG stream at an HRT of 3 days. However, at 1.0 kg m^–3^ day^–1^ fat loading rate, mixing of the floating fat layers became increasingly difficult for normal operation in both the SP and TP-MP reactors. Therefore, in SP, we divided the feeding into two streams—a daily pulse with concentrated FW and FOG mixture (28.0% fats) and continuous feeding of the remaining FW by a peristaltic pump. The TP-MP reactor was fed continuously with the content of the TP-AP reactor. The details about different fat feeding conditions in the TP-MP reactor are summarized in Table 2. To maintain an SRT of 100 days in the SP and TP-MP reactors, 52 mL sludge was wasted per day. The COD equivalent of the wasted sludge was calculated based on the concentration of total volatile solids (TVS) in the mixed liquor and using a conversion factor of 1.42 gCOD/gTVS. Similarly, biomass growth was estimated by monitoring the weekly increase in TVS concentration and the corresponding COD equivalent was calculated using the same conversion factor of 1.42 gCOD/gTVS. To clarify, this study is a continuation of our previous study (Amha et al., 2019) using the same inocula and same reactors. The co-digestion experiments were conducted immediately after the mono-digestion experiments ended without any gap in time.

**TABLE 2:** The operating conditions of the TP-MP reactor at different fat loading rates.

Target fats loading (kg m^–3^ day^–1^)	COD of the influent (g L^–1^) (FW+FOG)	Influent flow (L day^–1^)	HRT (days)	Flux (LMH)	Actual fat loading (kg m^–3^ day^–1^)
0.5	127.5	0.20	25.5	0.8	0.6
0.75	166.2	0.16	33.2	0.6	0.8
1	208.0	0.13	41.6	0.5	1.1

### Chemical Assays and Sampling

AnMBR performance was monitored by evaluating biogas production, biogas methane concentration, and effluent total COD concentration. Samples were filtered with 0.2-μm nylon membrane filters (Whatman, Pittsburgh, PA) to measure soluble constituents (e.g., ammonium, and different ions). The Nessler method (APHA, 2005) was used to measure ammonia concentrations, whereas ion chromatography (ICS-2000, Dionex, Sunnyvale, CA) was used to measure anions (nitrate and sulfate). Chromatographic separation was achieved using a 2-mm AS-11HC column (Dionex, Sunnyvale, CA). The composition of biogas was measured using a Trace 1310 GC system (Thermo Fisher Scientific, NY) equipped with a flame ionization detector. Hydrogen was used as the carrier gas, and a TG-BOND Q 30 m × 0.53 mm × 20 μm column was used for chromatographic separation. Total solids (TS) and total volatile solids (TVS) of the mixed liquor were determined using the procedures outlined in Standard Methods (APHA, 2005).

### Oxford Nanopore MinION Sequencing

Biomass samples collected weekly from the bulk were centrifuged at 5,000 g for 5 min at 4°C and decanted before storing at –80°C for further processing. For RNA preservation, biomass was stabilized using DNA/RNA shield (Zymo Research, Irvine, CA). Approximately 0.2 g of pelletized biomass was used for RNA extraction. RNA extraction was conducted using the Maxwell 16 simplyRNA kit according to the manufacturer’s instruction (Promega, Madison, WI). To eliminate DNA contamination, the extracted RNA was subjected to DNase treatment using DNA-free^TM^ DNA Removal Kit (Invitrogen, Carlsbad, CA). RNA concentration was measured using the Quant-iT RiboGreen RNA Assay (Invitrogen, Carlsbad, CA). Afterward, single-stranded complementary DNA (cDNA) was synthesized from the RNA samples using the GoScript^TM^ Reverse Transcription System according to the manufacturer’s instructions (Promega, Madison, WI). PCR of the cDNA samples was performed using universal 16S rRNA gene primers (i.e., 27F and 1492R) followed by gel electrophoresis to confirm the correct amplicon size (Klindworth et al., 2013). The PCR product was cleaned with 0.7× AMPure beads according to the manufacturer’s instruction (Beckman Coulter, Indianapolis, IN). The concentration of the cleaned DNA was measured spectrophotometrically using a BioSpectrometer (Eppendorf, Hamburg, Germany). Twenty ng of the clean DNA underwent a second round of PCR using MinION PCR barcodes according to the manufacturer’s instruction (Oxford Nanopore Technologies, United Kingdom). Gel electrophoresis was performed to confirm the expected amplicon size. Barcoded PCR products were cleaned using 0.7× AMPure beads, and DNA concentrations were measured as previously mentioned. Subsequent gel electrophoresis was performed in groups of four samples, using 150 ng of the barcoded DNA from each sample. The expected DNA band was extracted from the gel according to the manufacturer’s instruction (Roche Life Sciences, Germany). AMPure bead (0.7×) cleanup was performed, and the DNA concentration was measured as previously mentioned. The detail of the subsequent steps on self-ligation for the formation of a plasmid-like structure, rolling circle amplification (RCA), enzymatic de-branching, mechanical fragmentation, and DNA damage repair can be found in the Supplementary Text 1.

The end-repaired and dA-tailed RCA products were cleaned using regular AMPure beads (0.5× ratio) and eluted in 45 μL warm nuclease-free water. The DNA concentration was measured spectrophotometrically using a BioSpectrometer (Eppendorf, Hamburg, Germany), and the total yield of DNA was ∼ 900 ng. A 75-μL library was prepared using SQK-LSK109 and loaded onto a MinION MIN106 flow cell for 1D sequencing following the manufacturer’s instruction. The sequencing run continued for 24 h. FAST5 files from the MinKNOW software were base called using Guppy (version 3.4.4). The resultant FASTQ files were converted to FASTA files using Seqtk (Li, 2013). The FASTA files were then processed for consensus reads using INC-Seq, with a concatemer threshold of 4, as previously described (Li et al., 2016; Calus et al., 2018). The resultant INC-seq consensus reads were demultiplexed with Geneious Prime and known barcode sequences (Geneious Prime, 2020). Adaptor sequences from the consensus reads were trimmed using Porechop and size filtered for sequence lengths between 1,300 and 1,500 bp using Nanofilt (Jain et al., 2017; De Coster et al., 2018). The resultant reads were compiled into FASTA files for each respective time point using the command line cat (e.g., cat file1.fasta file2.fasta > combined.fasta). Chimeras were identified using UCHIME, and compiled FASTA files were preprocessed for operational taxonomic unit (OTU) clustering using Mothur version 1.43 (Schloss et al., 2009), with a similarity cutoff of 97%. Mothur and Silva release 132 (Quast et al., 2013) were used to classify OTUs at the genus level. Relative activity is defined as the ratio of 16S rRNA sequences (cDNA-based) for a given population to the total 16S rRNA sequences. Reverse transcription-quantitative PCR (RT-qPCR) of the 16S rRNA and *mcrA* transcripts were performed as described previously (Amha et al., 2017). Statistical analyses were conducted using JMP Pro (SAS Institute, NC). All raw sequences from this study are available in NCBI’s Sequence Read Archive (SRA) database (BioProject ID: PRJNA693521).

## Results and Discussion

### Performance of Single-Phase and Two-Phase AnMBRs at Different Fat Loading Rates

**FIGURE 1:**
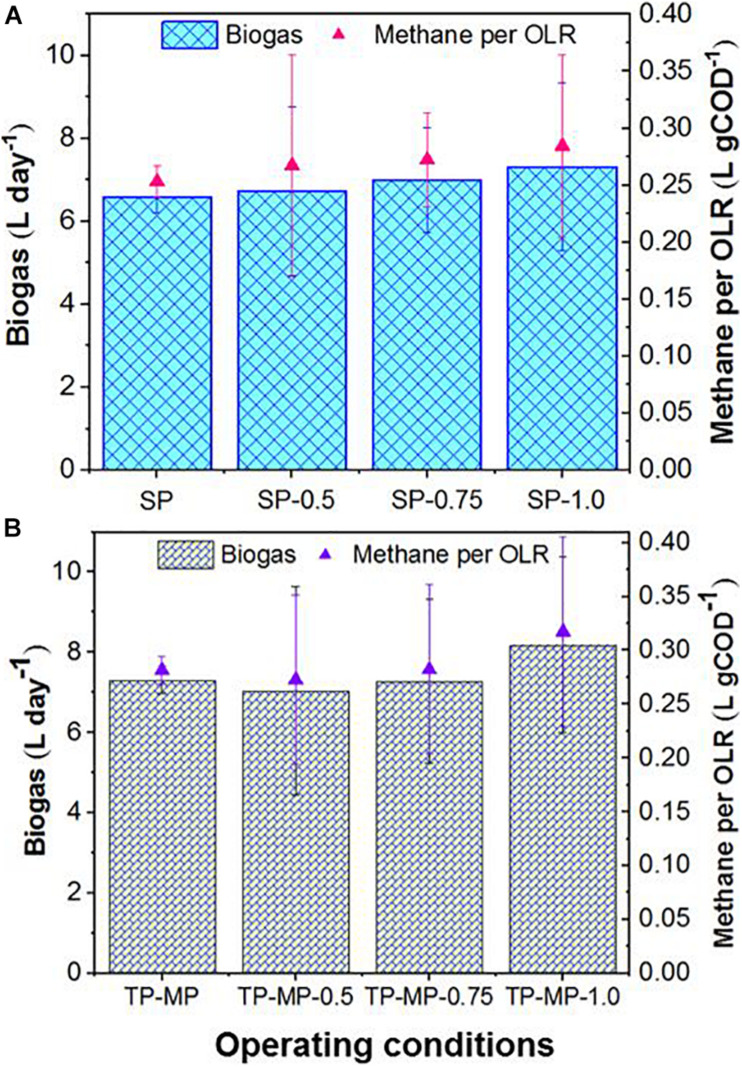
Biogas production rate and methane per OLR in single-phase **(A)** and two-phase **(B)** AnMBRs during mono and co-digestion of food waste and FOG. The number after SP and TP-MP shows the fat loading for each system. For example, TP-MP-0.5 means TP-MP AnMBR treatment at 0.5 kg m^–3^ day^–1^ fat loading. No number after SP and TP-MP means no fat addition with food waste (i.e., mono-digestion).

Increased biogas production was observed in the TP-MP reactor compared to the SP reactor at all fat loading rates; however, due to the high variability, the differences in biogas production rates were not statistically significant (Supplementary Figure 2). The highest increase of 12% biogas (7.3 ± 0.8 vs. 8.2 ± 0.8 L day^–1^) was observed at 1.0 kg m^–3^ day^–1^ fat loading in the TP-MP reactor compared to the SP reactor (Figure 1). A previous study on AnMBRs demonstrated a significant increase in biogas production in TP mode than in SP mode at an OLR of 3.5 gCOD L^–1^ day^–1^ and above (up to 10 gCOD L^–1^ day^–1^) when exclusively fed FW due to improved volatile fatty acid production in the AP reactor, and higher microbial diversity primarily due to syntrophic bacteria in the MP reactor (Amha et al., 2019). However, the co-digestion of FOG with food waste at higher OLR (i.e., 5 gCOD L^–1^ day^–1^) in the current study did not demonstrate such an increase. This could have resulted from FOG and/or long-chain fatty acid adsorption onto biomass surfaces which changed the cell morphology, decreased cell permeability, and limited the mass transfer of substrates and biogas (Galbraith and Miller, 1973; Pereira et al., 2005; Palatsi et al., 2009; Dereli et al., 2014). Although the decreased biomass activity due to LCFA accumulation can be reversed by intermittent feeding by slowly degrading the fat layer (Cavaleiro et al., 2008), we maintained continuous feeding in this study because severe inhibition was not observed in terms of biogas production as the fat loading increased.

**FIGURE 2:**
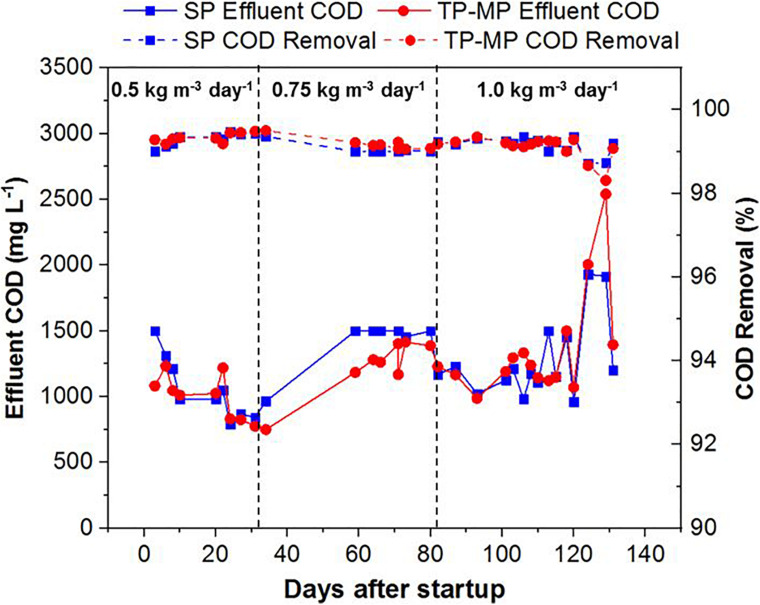
Chemical oxygen demand of the effluent and removal efficiency at different fat loading rates in both single-phase and two-phase methane-phase AnMBRs (each data point is the average of duplicate measurements).

Both the SP and TP AnMBRs demonstrated high effluent quality with the COD removal ranging from 98.7 to 99.5% for SP and 98.3 to 99.5% for TP-MP (Figure 2). The high COD removal is attributed to both organic degradation and membrane separation in the reactor (Smith et al., 2012; Lin H. et al., 2013). The effluent COD was comparatively higher at 0.75 and 1.0 kg m^–3^ day^–1^ fat loading after 120 days (Figure 2), likely because of the inhibiting effect of LCFA on the biomass (Chen et al., 2008; Sousa et al., 2013). However, the COD reduction was still above 98% during that period. The COD mass balance indicated that most of the COD was converted to methane and waste biomass (Supplementary Figure 3), which agrees with our previous study (Amha et al., 2019). Because of the slow growth of anaerobic microorganisms, the COD utilized for biomass growth was lower than the COD utilized for methane production and COD wasted as biomass (Strous et al., 1998; Riviere et al., 2009). The pH remained stable throughout this study in SP, TP-MP, TP-AP1, and TP-AP2, with an average value of 7.9 ± 0.2, 8.0 ± 0.2, 3.8 ± 0.2, and 3.3 ± 0.1, respectively (Supplementary Figure 4), which are within the optimum pH for anaerobic and acid-phase digestion (Demirel and Yenigün, 2002; Zhang et al., 2014). Total ammonia nitrogen concentrations also remained stable throughout operation, with concentrations of 1.27 ± 0.17 g L^–1^ in SP and 1.62 ± 0.16 g L^–1^ in TP-MP with a removal ranging from 15.6 to 33.8%, attributed to microbial uptake and anaerobic degradation of ammonia (Yenigün and Demirel, 2013; Krakat et al., 2017). The corresponding free ammonia nitrogen was 64 and 100 mg L^–1^, respectively. The inhibitory free ammonia concentration varies among studies; however, free ammonia concentration as low as 80 mg L^–1^ can cause inhibition during anaerobic digestion (Chen et al., 2008; Werner et al., 2014; Amha et al., 2018). Thus, the free ammonia concentration in our study likely only had a minor inhibitory effect.

### Comparison of Mono-digestion of FW With Co-digestion of FW and FOG

The biogas production rate for co-digestion of FW and FOG was significantly higher (*p* < 0.05) than mono-digestion of FW at 1.0 kg m^–3^ day^–1^ fat loading rate for both SP and TP AnMBRs (Figure 1). In TP-MP, the biogas production rate was 13.2% higher in co-digestion than mono-digestion at 1.0 kg m^–3^ day^–1^ fat feeding condition. Similarly, in SP AnMBR, the biogas production rate was significantly higher in co-digestion than in mono-digestion at both 0.75 (6.4% higher) and 1.0 kg m^–3^ day^–1^ (11.3% higher) fat loading rates. These results indicate that co-digestion at low fat loading rates (0.5–0.75 kg m^–3^ day^–1^) does not significantly increase the biogas production rate in TP reactors compared to mono-digestion. However, at a low fat loading rate (i.e., 0.75 kg m^–3^ day^–1^), SP co-digestion significantly increased biogas production relative to mono-digestion. Methane content was consistently in the range of 60–70% for the lower fat loading rates (i.e., 0.5 and 0.75 kg m^–3^ day^–1^), whereas it was marginally higher (up to 74%) for the 1.0-kg m^–3^ day^–1^ fat loading rate (Supplementary Figure 2). This may have resulted from the high convertibility (94.8%) of FOG lipids to biogas compared to carbohydrates (50.4%) and proteins (71%) of other substrates (Jeganathan et al., 2006; Davidsson et al., 2008; Ziels et al., 2016). In general, the observed methane per OLR was higher during co-digestion than during mono-digestion and the difference was significant (*p* < 0.05) at a fat loading of 1.0 kg m^–3^ day^–1^ (Figure 1). Further, methane per OLR was higher (*p* > 0.05) for TP-MP compared to SP for all fat loading rates, reaching the highest value of 0.32 L gCOD^–1^ at a 1.0-kg m^–3^ day^–1^ fat loading rate (Figure 1). The specific methane yield in this study is slightly higher than a previous AnMBR study treating a lipid-rich corn-to-ethanol thin stillage at OLRs up to 8.0 gCOD L^–1^ d^–1^ (Dereli et al., 2014). The increasing specific methane yield observed in our research with the increase in fat loading rate suggests no inhibition as the fat loading rate was increased from 0.5 to 1.0 kg m^–3^ day^–1^, although the higher effluent COD at 0.75 and 1.0 kg m^–3^ day^–1^ fat loading compared to the 0.5-kg m^–3^ day^–1^ fat loading indicates minor inhibition. The biomass at 1.0 kg m^–3^ day^–1^ demonstrated extreme sensitivity toward an accidental exposure to oxygen for 3–5 h. The reactors were unable to be revived after ensuring completely anaerobic conditions for more than 2 weeks.

### Microbial Community Analysis

**FIGURE 3:**
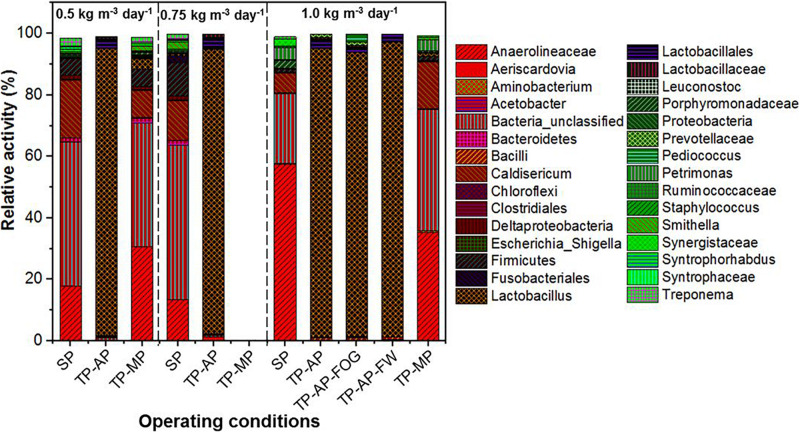
Relative activity based on 16S rRNA sequencing identified at the genus level where possible for different fat loadings and operating conditions. All data are expressed as a percentage of total 16S rRNA sequences (bacteria) per sample point. Due to the accidental loss of extracted RNA from TP-MP-0.75 samples, no corresponding data is presented here.

MinION sequencing of the reverse-transcribed 16S rRNA revealed a dominance of fermenters, fatty acid degraders, and unclassified bacteria in the biomass samples from different operating conditions (Figure 3). While we acknowledge that methanogenic archaea are important members of the microbial population in AnMBR systems, there are currently no established universal full-length 16S rRNA primers designed to detect both Bacteria and Archaea. Thus, this study used universal full-length 16S rRNA primers designed for the Bacteria domain. *Lactobacillus*, capable of fermentative metabolism, dominated in the AP reactors with a genus-level relative activity of 92.2–95.7%, which is consistent with reports of *Lactobacillus* surviving in low pH conditions (Rault et al., 2009). The relative activity of *Lactobacillus* in the co-digestion mode in all fat loading rates was much higher than the 72–82% relative activity of *Lactobacillus* in the mono-digestion mode (Amha et al., 2019). The similar relative activity of *Lactobacillus* in the AP reactors could be because of the high relative activity (78.0–86.0%) of *Lactobacillus* in the raw food waste used in this study (Amha et al., 2019). This is consistent with previous observations showing dominant *Lactobacillus* relative activity in food waste and anaerobic digesters (Shin et al., 2010). In the SP and TP-MP reactors, *Anaerolineaceae* showed high relative activity ranging from 13.3 to 57.5%. This relative activity of *Anaerolineaceae* in the co-digestion mode is much higher than the relative activity in the mono-digestion mode at the same OLR (Amha et al., 2019). *Anaerolineaceae* are fermenters that can degrade alkanes in oil and hydrocarbon environments and are known to form a syntrophic relationship with *Methanosaeta* during methanogenic degradation of alkanes (Liang et al., 2015; McIlroy et al., 2017). *Firmicutes* were found in low relative activity in all samples, with the highest relative activity detected in the SP reactor samples (1.2–10.9%), while *Bacteroidetes* were mainly found in the reactors at the 0.5-kg m^–3^ day^–1^ fat loading rate. Both *Firmicutes* and *Bacteroidetes* can cause the anaerobic hydrolysis and acidification of FOG (Sousa et al., 2007a; Yang et al., 2016; Ferguson et al., 2018).

**FIGURE 4:**
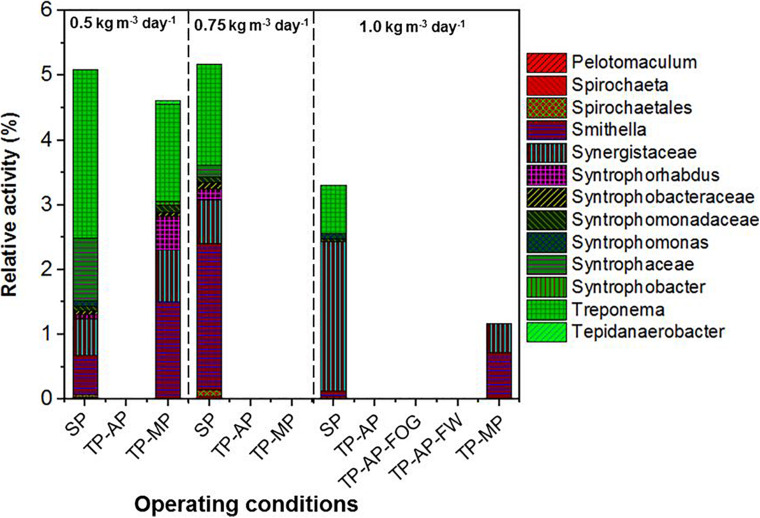
Relative activity of syntrophic fatty acid oxidizers based on 16S rRNA sequencing identified at the genus level for different fat loadings and operating conditions. All data are expressed as a percentage of total 16S rRNA sequences (bacteria) per sample point.

Higher relative activity of *Syntrophomonas*, *Syntrophobacteraceae*, *Syntrophaceae*, and other syntrophic fatty acid oxidizers were observed in the SP and TP-MP reactors than the AP reactors (Figure 4). This observation is due to the low pH conditons and low SRT of the AP, which inhibits methanogens and their syntrophic counterparts (Xu et al., 2014). It is reported that unsaturated and saturated LCFA-oxidizing bacteria are from *Syntrophomonadaceae* and *Syntrophobacteraceae* families (Sousa et al., 2007b; Salama et al., 2019). *Syntrophomonas wolfei* is a saturated short/medium-chain fatty acid degrader, and *Syntrophomonas zehnderi* is a saturated and unsaturated LCFA degrader (Sousa et al., 2007b). *Syntrophomonas sapovorans*, *Syntrophomonas curvata*, and *Syntrophomonas zehnderi* can utilize mono- and/or polyunsaturated LCFAs (Salama et al., 2019). *Treponema* were found in low relative activity (<2.7%) in the SP samples for all fat loading rates, and only in the TP-MP samples at the 0.5-kg m^–3^ day^–1^ fat loading rate. *Treponema* are fermenters and fatty-acid degraders that can survive in high ammonia (10 g L^–1^) environments and degrade polysaccharides and disaccharides (Paster and Canaleparola, 1985; Poirier et al., 2016). Unclassified Bacteria dominated in the SP and TP-MP reactors at high relative activities at all fat loadings, ranging from 22.7 to 50.3%. This indicates that unclassfied bacteria are an important part of these systems during FW and FOG co-digestion (Amha et al., 2019).

**FIGURE 5:**
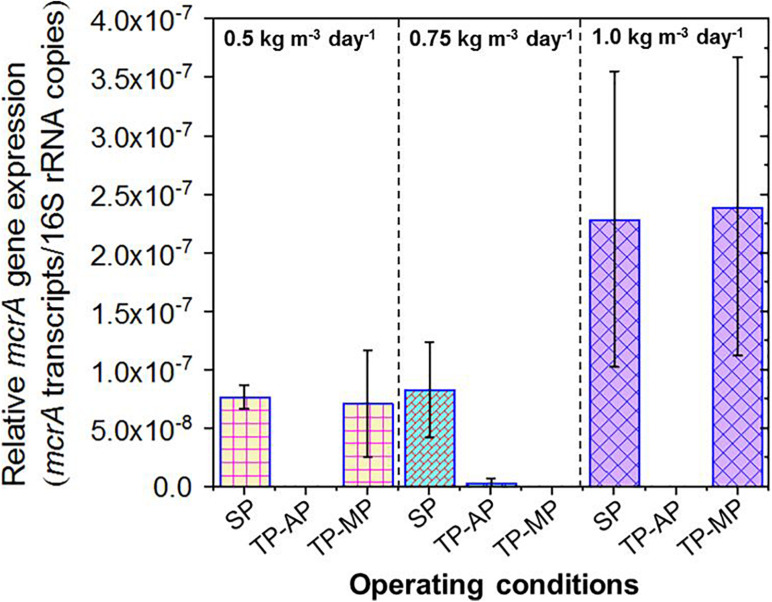
Relative expression of *mcrA* genes in the samples from all operating conditions. Copies of *mcrA* transcripts were normalized to total 16S rRNA copies. Due to the accidental loss of extracted RNA from TP-MP-0.75 samples, no corresponding data is presented here.

Due to the lack of 16S rRNA data for methanogens, we instead relied on quantification of *mcrA* gene expression as an aggregate measurement of methanogenic activity. The *mcrA* to 16S rRNA ratio was significantly higher for both the SP and TP-MP conditions compared to the AP conditions in all cases (Figure 5). This is likely due to the greater abundance of the *mcrA* gene copies (and methanogens) in the SP and MP compared to the AP. The relative *mcrA* gene expression was higher at the 1.0-kg m^–3^ day^–1^ fat loading for both the SP (*p* = 0.28) and TP-MP (*p* = 0.08) conditions compared to the 0.5-kg m^–3^ day^–1^ fat loading. This corroborates the positive correlation of mcrA gene abundance with methane production.

## Conclusion

This study demonstrated a statistically insignificant increase of biogas production in the TP AnMBR compared to the SP AnMBR during co-digestion of FW and FOG at different fat loading rates. However, a significant increase in biogas production was observed in the co-digestion mode in both SP and TP AnMBRs at the 1.0-kg m^–3^ day^–1^ fat loading rate than the mono-digestion of FW only. This indicates the applicability of AnMBR at high fat loading rates without any major inhibition. Third-generation MinION sequencing identified the predominance of fermenters and fatty-acid degraders in the AnMBRs, which are vital for the improved performance in synergistic cooperation with methanogens. Overall, this study demonstrated that FOG co-digestion with FW could be an effective operational strategy to improve the performance of AnMBRs treating FW.

## Data Availability Statement

The datasets generated for this study can be found in the online repositories. The name of the repository and accession number can be found below: National Center for Biotechnology Information (NCBI), https://www.ncbi.nlm.nih.gov/, PRJNA693521.

## Author Contributions

SI and YA led the project and manuscript preparation. PW led the MinION sequencing part. QD and JL helped with the experiment and the manuscript. MC provided valuable insight into the manuscript. AS supervised the whole project. All authors contributed to the article and approved the submitted version.

## Conflict of Interest

YA was employed by Trussell Technologies, Inc., and MC was employed by Divert, Inc. The remaining authors declare that the research was conducted in the absence of any commercial or financial relationships that could be construed as a potential conflict of interest.
