# Supplementary material for: Investigation of Fats, Oils, and Grease Co-digestion With Food Waste in Anaerobic Membrane Bioreactors and the Associated Microbial Community Using MinION Sequencing
Source: Front Bioeng Biotechnol. 2021 Apr 12;9:613626. doi: 10.3389/fbioe.2021.613626 (PMC8072289; doi:10.3389/fbioe.2021.613626)
Supplement: Supplementary file 1 [file Data_Sheet_1.docx]

**Investigation offats, oils, and greaseco-digestion with food waste in anaerobic membrane bioreactorsand the associated microbial community using MinION sequencing**

Syeed Md Iskander^a^, Yamrot M. Amha^b^, Phillip Wang^a^, Qin Dong^a^, Juhe Liu^a^, Michael Corbett^c^, and Adam L. Smith^a^

^a^ Astani Department of Civil and Environmental Engineering, University of Southern California, 3620 South Vermont Avenue, Los Angeles, CA 90089, USA

^b^ Trussell Technologies, Inc., 232 N Lake Ave, Pasadena, CA 91101, USA

^c^ Divert, Inc., 23 Bradford Street, 3rd Floor, Concord, Massachusetts 01742, USA

†Corresponding author (Adam L. Smith)

Phone: +1 213.740.0473

Email: [smithada@usc.edu](mailto:smithada@usc.edu)

**Text S1**

In this stage 5 µL of Blunt/TA Ligase Master Mix (NEB, Ipswich, MA) was added with 45 µL of the DNA sample to circularize the linear amplicons into plasmid-like structure. The mixture was incubated at 10°C for 15 minutes and 25°C for 10 minutes. The linear amplicons that did not self-ligate into circular plasmid-like structure, but formed long chimeric linear amplicons, were removed using AMPure beads cleanup. Before cleanup, 150 µL completely mixed beads were concentrated by 50% by removing the buffer. 17.5 µL (0.35x ratio) of these concentrated beads was added to the 50 µL post-ligation reaction mixture to remove the amplicons greater than 2,000 bp. At first, the mixed beads and the reaction mix was incubated for 2 min at room temperature in a 2 mL tube which was then placed in a magnetic rack. The beads bound to the long amplicons and the short amplicons (less than 2000 bp) remained in the solution, which (67.5 µL) was separated in a 2 mL tube. The short amplicons were cleaned up again using standard AMPure beads (0.5x ratio) following the manufacturer’s instruction and eluted in 15 µL nuclease free water. To digest the linear amplicons from the plasmid mix, the mixture was treated with Plasmid-Safe DNase following the mini-prep protocol according to the manufacturer’s instruction (Lucigen, Middleton, WI). Rolling circle amplification (RCA) of the plasmid pool was performed using TruePrime^TM^ RCA kit according the manufacturer’s instructions. The reaction was performed in triplicates with an additional negative control without any plasmid. The reaction mix was incubated at 29.5 °C for 150 minutes and the DNA concentration was measured using Quant-iT PicoGreen dsDNA Assay kit (Invitrogen, Carlsbad, CA).

The RCA product had a final concentration range of20 – 30 ng µL^-1^ and no unspecific product was formed in the negative control. To remove hyperbranching structures formed during RCA, triplicate RCA product wasmixed and debranched by treating with T7 endonuclease I enzyme(NEB, Ipswich, MA). For this,2 µL of the T7 endonuclease I enzyme was mixed with 63 µL of the RCA product followed by 5 min incubation at room temperature. Mechanical fragmentation of the debranched RCA product was performed using g-TUBE (Covaris, Woburn, MA) by centrifuging at 4000 rpm until the entire reaction mix passed through the hole. The tube was then reversed to repeat the centrifugation process. The fragmented RCA product was cleaned using previously prepared 50% concentrated beads at 0.35x ratio according to the manufacturer’s instructions. The amplicons bound to the beads were eluted using 65 µL of nuclease-free warm water. The secondary enzymatic de-branching was performed to remove hyper-branched structures completely. For this, 2 µL of the T7 endonuclease I enzyme was mixed with 63 µL of the RCA product and incubated at 37 ºC for 5 min. This was followed by another 50% concentrated AMpure beads clean up at 0.45x ratio and elution at 55 µL warm nuclease free water. The final de-branched RCA product was treated with NEBNextFFPE DNA Repair Mix (NEB, Ipswich, MA) for gap-filling and dA-tailing caused during g-TUBE fragmentation and T7 endonuclease I enzyme treatment. The reaction mix was prepared according to the manufacturer’s instruction and incubated at 20 ºC for 30 min followed by another 30 min at 60 ºC. The end-repaired and dA-tailed RCA product was cleaned using regular AMPure beads and went through library preparation for 1D MinION sequencing.

**Table S1.**Operating conditions at different fats loading rates.

| Target fats loading (kg m^-3^ day^-1^) | COD of the influent (g L^-1^) (FW+FOG) | Influent flow (L day^-1^) | HRT (days) | Flux (LMH) | Actual fats loading (kg m^-3^ day^-1^) |
| --- | --- | --- | --- | --- | --- |
| 0.5 | 127.5 | 0.20 | 25.5 | 0.8 | 0.6 |
| 0.75 | 166.2 | 0.16 | 33.2 | 0.6 | 0.8 |
| 1 | 208.0 | 0.13 | 41.6 | 0.5 | 1.1 |


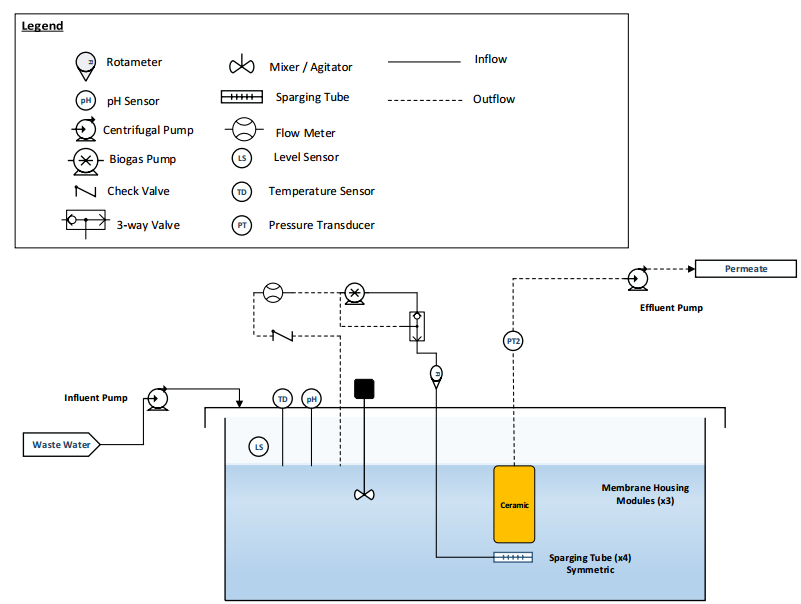


**Figure S1.**The schematic of a bench-scale AnMBR.


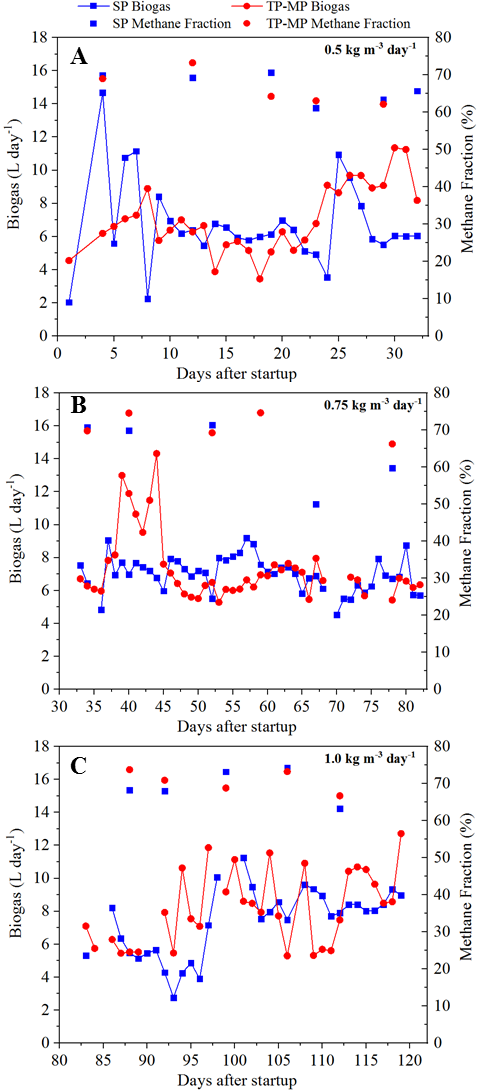


**Figure S2.** Daily biogas production rate and biogas methane fraction at (A) 0.5 kg m^-3^day^-1^, (B) 0.75 kg m^-3^day^-1^, and (C) 1.0 kg m^-3^day^-1^ fats loading rates in single phase (SP) and two-phase methane phase (TP-MP) AnMBRs.


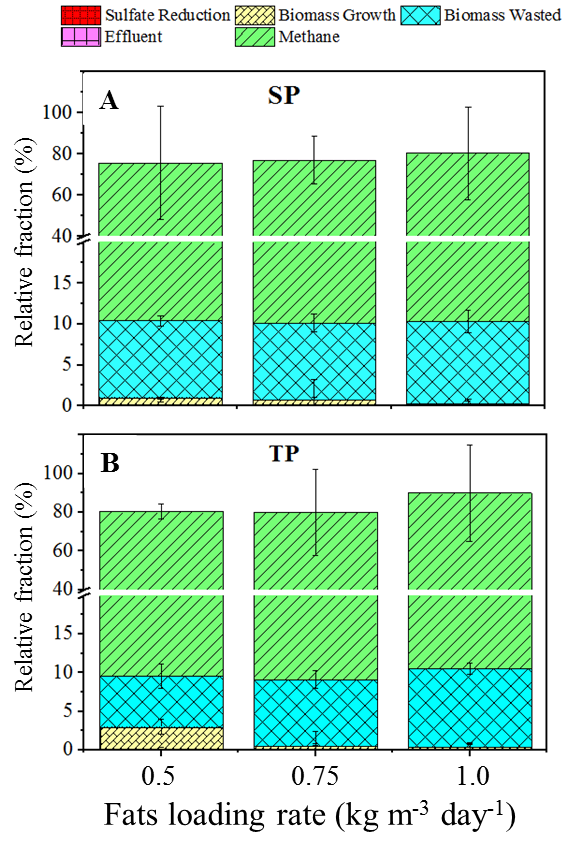


**Figure S3.** (A) Mass balance analysis for SP based on COD allocation of output relative to input (%), at different fats addition. (B) Mass balance analysis for TP-MP based on COD allocation of output relative to input (%), at different fats addition. Complete sulfate reduction was assumed based on influent sulfate concentration.


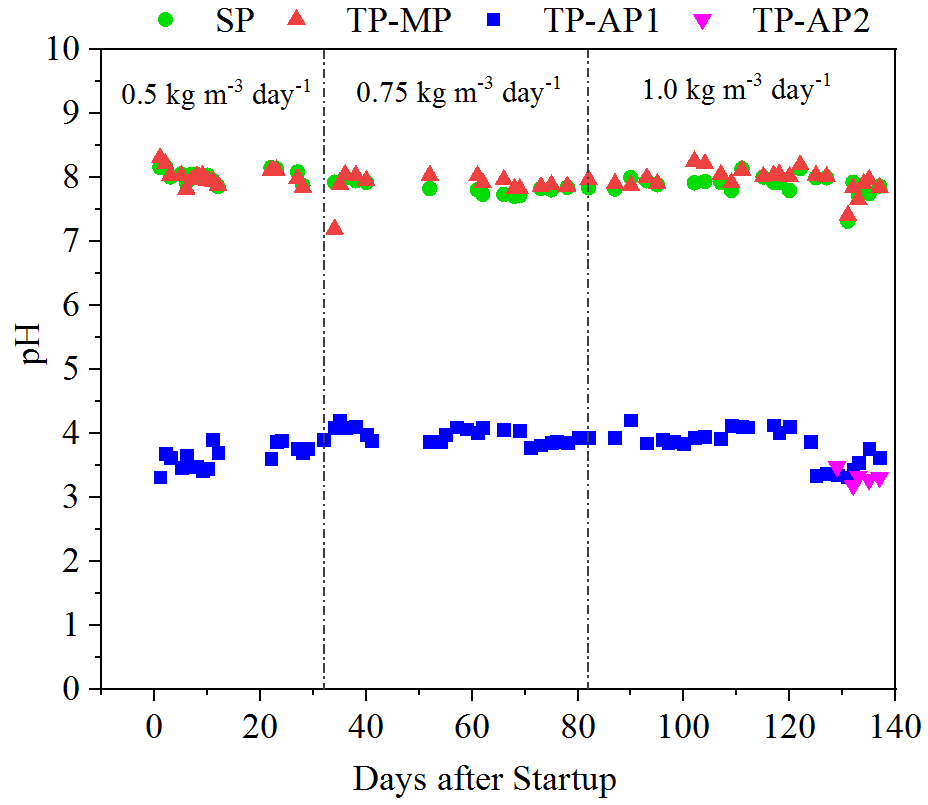


**Figure S4.** pH in the single phase(SP), two-phase methane phase (TP-MP), andtwo-phase acid phase (TP-AP1 and TP-AP2) reactors at different fats loading rates.TP-AP1 was fed with FW only, and TP-AP2 with concentrated FW and FOG mixture.
